# Atrial Fibrillation Increases Proarrhythmic Mechanisms in the Ventricle

**DOI:** 10.1016/j.jacbts.2026.101558

**Published:** 2026-05-07

**Authors:** Paul Spangler, Thea Bommer, Laura Stengel, Petros Tirilomis, Thomas Körtl, Laura M. Schreiner, Thomas Sowa, Tobias Uhe, Christof Schmid, Zdenek Provaznik, Theodor Tirilomis, Rolf Wachter, Aneesh Bapat, Matthias Nahrendorf, Lars S. Maier, Stefan Wagner, Samuel Sossalla, Steffen Pabel

**Affiliations:** a Department of Internal Medicine II, University Medical Center Regensburg, Germany; b Department of Cardiology, University Hospital Giessen, Kerckhoff Clinic Bad Nauheim, and DZHK, Partner site RhineMain, Germany; c Clinic for Cardiology and Pneumology, University Medical Center Göttingen, and DZHK (German Centre for Cardiovascular Research), partner site Göttingen, Germany; d Department of Cardiology, University Hospital Leipzig, Germany; e Department of Cardiothoracic Surgery, University Medical Center Regensburg, Germany; f Department for Thoracic, Cardiac, and Vascular Surgery, University Medical Center Göttingen, Germany; g Cardiovascular Research Center, Massachusetts General Hospital, Boston, Massachusetts, USA; h Demoulas Cardiac Arrhythmia Service, Division of Cardiovascular Medicine, Department of Medicine, Massachusetts General Hospital, Boston, Massachusetts, USA; i Center for Systems Biology, Massachusetts General Hospital and Harvard Medical School, Boston, Massachusetts, USA

**Keywords:** atrial fibrillation, Ca^2+^ homeostasis, excitation-contraction coupling, ventricular arrhythmias, translational studies

## Abstract

Ventricular arrhythmias and sudden cardiac death are a leading cause of death in patients with atrial fibrillation (AF). This study investigated how AF promotes ventricular arrhythmias.

Cellular electrophysiology of AF patients was characterized using human left ventricular (LV) samples from patients with sinus rhythm (n = 25) or rate-controlled AF (n = 16). In LV cardiomyocytes from AF patients, delayed afterdepolarizations and Ca^2+^ waves, both established triggers for ventricular arrhythmias, were significantly more frequent compared to sinus rhythm patients. In vitro AF simulation in murine LV cardiomyocytes confirmed increased ventricular proarrhythmic activity upon AF. Oxidative Ca^2+^/calmodulin-dependent protein kinase II activation was increased in murine LV cardiomyocytes after AF simulation. MMVV knock-in mice with genetic ablation of Ca^2+^/calmodulin-dependent protein kinase II oxidation did not develop ventricular proarrhythmic activity after AF simulation. This study demonstrates that AF promotes ventricular arrhythmogenesis. The findings offer new mechanistic insights into the interaction of AF and ventricular arrhythmias.

## Highlights

• AF is associated with ventricular arrhythmic triggers: ventricular cardiomyocytes from patients with AF exhibited increased diastolic Ca^2+^ waves and delayed afterdepolarizations compared with sinus rhythm controls.
• Irregular ventricular activation alone induces proarrhythmic mechanisms: in murine ventricular cardiomyocytes, AF simulation with irregular pacing induced arrhythmic events.
• Oxidative CaMKII activation and NCX mediate AF-induced arrhythmogenesis: AF simulation increased CaMKII oxidation and phosphorylation of Ca^2+^-handling proteins, promoted diastolic Ca^2+^-release, and increased NCX activity, facilitating NCX-mediated triggered activity.
• Genetic ablation of CaMKII oxidation prevents AF-induced ventricular arrhythmogenesis: cardiomyocytes lacking regulatory CaMKII oxidation sites were protected from AF-induced arrhythmogenic Ca^2+^-release events.
• These findings identify a mechanistic link between AF and ventricular arrhythmias.

## Visual Abstract

**Figure:**
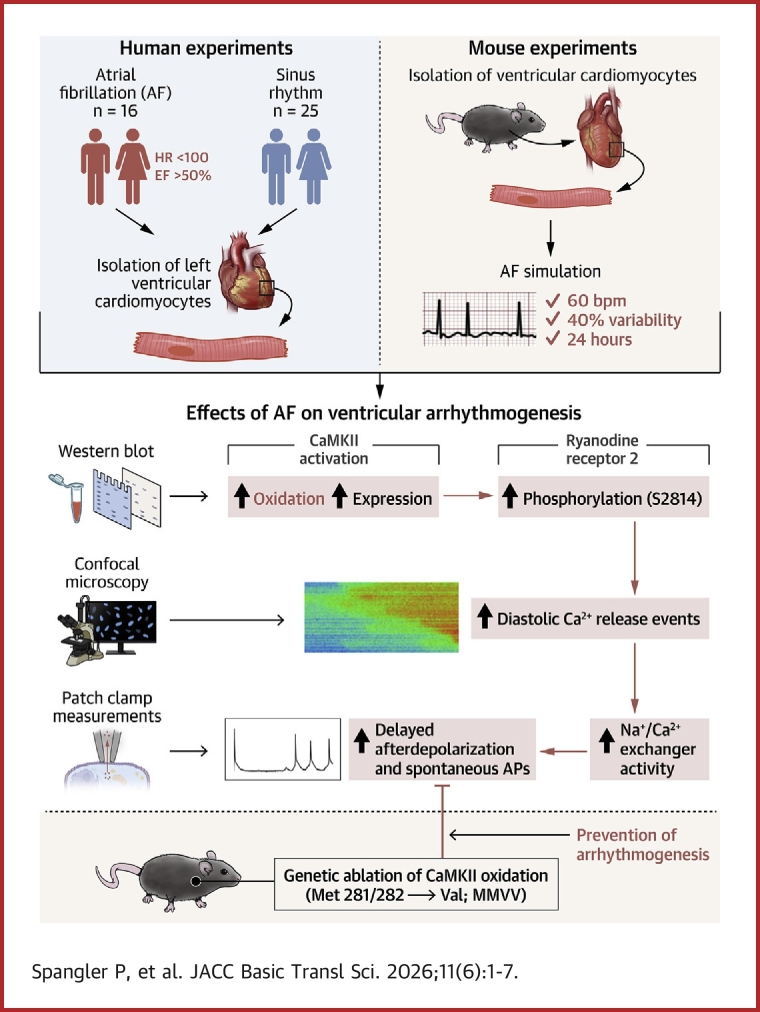

Atrial fibrillation (AF) is the most prevalent arrhythmia worldwide, currently affecting more than 33 million individuals, with its global burden expected to increase 2- to 3-fold by 2050.^1^ Although clinical management of patients with AF focuses on the prevention of thromboembolic events and heart failure (HF), sudden cardiac death (SCD) represents the leading cause of death in patients with AF.^2^ Clinical studies, such as the ARIC (Atherosclerosis Risk in Communities) study, have shown an independent and significant association between AF and SCD, as well as AF and ventricular fibrillation—the leading immediate cause of SCD in the general population.^3^^,^^4^ Despite this prognostic significance, and in contrast to the well-established causal link between AF and stroke, the mechanisms by which AF predisposes to ventricular arrhythmias remain poorly understood.

AF has predominantly been perceived as an atrial disorder, and as a result, AF research mainly focused on the atria. Thus, the consequences of AF on left ventricular (LV) function have remained largely underexplored. Clinical evidence suggests that AF may exert deleterious effects on LV function, increase mortality, and exacerbate HF-related morbidity and mortality in patients with HF.^5^, ^6^, ^7^ The EAST-AFNET 4 (Early Treatment of Atrial Fibrillation for Stroke Prevention) study showed that early rhythm control, as opposed to rate control alone, led to improved cardiovascular outcomes in patients with recent-onset AF and a cardiovascular condition.^8^ These findings underscore the translational importance of investigating the mechanisms of how even rate-controlled AF may affect the LV.

Although AF is associated with increased atrial fibrosis, studies of LV fibrosis have indicated no difference between patients with AF and those in sinus rhythm (SR).^9^^,^^10^ This lack of structural remodeling in ventricles of patients with AF suggests that electrophysiologic remodeling may underlie the deleterious effects of AF on the LV. In our previous work, we demonstrated that AF itself impairs LV function through adverse remodeling of cardiomyocyte excitation–contraction coupling.^9^ Importantly, altered Na^+^ and Ca^2+^ homeostasis have been implicated in ventricular arrhythmogenesis.^11^

This translational study aimed to investigate whether AF promotes ventricular arrhythmias. We studied the impact of AF on ventricular arrhythmogenesis by using several electrophysiological techniques to analyze cardiomyocyte electrophysiology in human ventricular myocardium. We studied patients with preserved LV function who underwent surgical aortic valve replacement for aortic stenosis (AS), presenting with rate-controlled permanent or persistent AF or SR. Considering potential confounders present in patient cohorts, such as comorbidities and medication, we further examined the effects and mechanisms of rate-controlled AF on ventricular arrhythmogenesis in murine ventricular cardiomyocytes by performing an in vitro AF simulation.^9^^,^^12^

## Methods

### Patients

All procedures in this study were conducted in accordance with the ethical standards outlined in the Declaration of Helsinki and were approved by the local Ethics Committee of the University of Regensburg (reference number 31/9/00) and the University of Göttingen (reference numbers 10/9/15, 21/10/00 and 21/2/11). Written informed consent was obtained from all participating patients. LV myocardial tissue was obtained from individuals undergoing surgical aortic valve replacement for AS. The study included patients with SR as well as clinically diagnosed permanent or persistent rate-controlled AF. LV tissue was excised from the septum and immediately placed into cooled cardioplegic solution (4 °C) for transportation. Detailed patient characteristics are provided in Table 1.

**Table 1:** Clinical Characteristics of Patients With SR and AF

	SR (n = 25)	AF (n = 16)	*P* Value
Demographics			
Male (%)	52.0 (13/25)	81.3 (13/16)	0.058
Age (y)	66.6 ± 8.2 (25)	69.2 ± 15.6 (16)	0.48
BMI, kg/m^2^	29.5 ± 5.3 (15)	29.6 ± 5.3 (10)	0.99
White (%)	100.0 (25/25)	100.0 (16/16)	0.99
Hemodynamics and imaging			
Heart rate, beats/min	65.0 (64.0-71.0; 15)	71.5 (65.0-83.8; 10)	0.11
Ejection fraction, %	59.0 (54.5-60.0; 25)	60 (50.0-60.0; 15)	0.98
IVS, mm	13.0 (12.0-15.0; 21)	14.0 (12.0-14.0; 11)	0.76
AVA, cm^2^	0.80 (0.70-0.90; 21)	0.70 (0.60-0.90; 15)	0.24
AV mean gradient, mm Hg	39.2 ± 18.7 (21)	34.6 ± 11.4 (11)	0.46
LVEDD, mm	47.1 ± 6.2 (19)	54.9 ± 8.5 (11)	0.007^a^
Comorbidities			
Coronary artery disease, %	43.5 (10/23)	62.5 (10/16)	0.24
Diabetes, %	20.8 (5/24)	43.8 (7/16)	0.12
GFR, mL/min	65.9 ± 27.0 (14)	51.0 ± 13.1 (8)	0.16
Medication			
Insulin, %	21.4 (3/14)	40.0 (4/10)	0.32
Antidiabetics, %	21.4 (3/14)	10.0 (1/10)	0.46
ACE inhibitor or ARB, %	40.9 (9/22)	43.8 (7/16)	0.86
β-blocker, %	63.6 (14/22)	62.5 (10/16)	0.94
Aldosterone antagonist, %	9.10 (2/22)	13.3 (2/15)	0.68
Diuretic, %	59.1 (13/22)	66.7 (10/15)	0.64
Digitalis, %	0.0 (0/22)	16.7 (2/12)	0.12
Antiarrhythmic, %	0.0 (0/22)	0.0 (0/16)	0.99
Statins, %	60.9 (14/23)	62.5 (10/16)	0.92

Values are % (n/N), mean ± SD (n), or median (Q1-Q3; n). Myocardial tissue was obtained during surgical aortic valve replacement. Comparisons between groups were performed using Student’s *t*-test for parametric data, the Mann–Whitney U test (unpaired) for nonparametric data, and the chi square test or Fisher exact test for categorical variables, as appropriate. Complete clinical data were not available for every patient.

ACE = angiotensin-converting enzyme; AF = atrial fibrillation; ARB = angiotensin II receptor blocker; AV = aortic valve; AVA = aortic valve area; BMI = body mass index; GFR = glomerular filtration rate; IVS = interventricular septum; LVEDD = left ventricular end-diastolic diameter; SR = sinus rhythm.

a Indicate statistical significance: *P* < 0.05.

### Isolation of human LV cardiomyocytes

Ventricular myocardium was meticulously cleared of fibrotic tissue while being immersed in cardioplegic solution. The choice of cardiomyocyte isolation method was determined by the size and integrity of the available myocardial specimen. For larger tissue samples (>50-100 mg), a conventional chunk isolation approach was applied, whereas tissue slicing–assisted digestion (TSAD) was preferentially used for small myocardial samples (<30-50 mg) or thin surgical remnants with limited volume. Chunk isolation was performed as described previously.^9^ In brief, tissue was enzymatically digested using a 2-step collagenase/proteinase protocol in Joklik–minimum essential medium buffer. After initial digestion (23 minutes) with 0.9 to 0.95 mg/mL of collagenase (Worthington type I, 315 U/mg), 0.23 mg/mL of proteinase (Sigma-Aldrich, 7-14 U/mg), and 2 mg/mL of bovine serum albumin (BSA; Sigma-Aldrich), a second digestion step (5-10 minutes) with adjusted enzyme concentrations containing 0.58 mg/mL of collagenase and 2.67 mg/mL of BSA followed. Liberated cells were collected, and residual tissue was mechanically dissociated. Cells were washed and resuspended in Joklik- minimum essential medium solution containing 5 mg/mL of BSA. This process was repeated 5 to 8 times to maximize yield. Chunk isolation enables simultaneous enzymatic digestion of larger myocardial pieces and is therefore advantageous when sufficient tissue mass is available. Using the chunk isolation approach, higher absolute cell yields were obtained, corresponding to approximately 80 to 150 cardiomyocytes per milligram tissue, depending on sample quality and enzymatic efficiency.^9^^,^^13^^,^^14^

For TSAD, myocardial samples were embedded in 4% low-melting-point agarose (Carl Roth), cooled, and mounted onto a vibratome specimen holder using a thin layer of cyanoacrylate glue. To maximize the surface-to-volume ratio while minimizing cellular damage, 300-μm slices were generated using a vibratome (Leica Biosystems) and consequently stored in a glucose-free slicing buffer before they were gently detached from the agarose and transferred to a culture dish containing washing solution. A stepwise and controlled enzymatic digestion protocol was applied, including intermediate washing steps between enzymatic exposures. Initially, tissue slices were incubated for 10 minutes in a solution containing 1 mg/mL protease, followed by digestion with 4 mg/mL collagenase. During the collagenase phase, the dish was regularly observed under the microscope to monitor myocyte release. Once the tissue exhibited visible softening and could be dissociated with gentle mechanical manipulation, digestion was halted by adding a stop solution containing 10 mg/mL of BSA. Tissue dissociation was performed using fine forceps to gently tease apart myocardial fibers. TSAD was developed to facilitate cardiomyocyte isolation from small or limited human myocardial samples by increasing the surface-to-volume ratio and enabling more homogeneous enzyme penetration. Although TSAD typically yields fewer cells (ranging from 9-100 cells per milligram) compared with chunk isolation, it provides a reliable alternative when tissue availability is limited and ensures reproducible isolation under such conditions.^15^

Cell quality was assessed using established morphological criteria, including rod-shaped appearance, clear cross-striations, and absence of spontaneous contracture or hypercontracture. Previous studies have shown that slicing-based isolation approaches, including TSAD, yield cardiomyocytes with preserved structural and functional properties comparable to conventional chunk isolation, albeit at lower absolute cell numbers. Based on these considerations, chunk isolation was used when the myocardial sample size exceeded 50 mg, whereas TSAD was used for smaller specimens below this threshold, ensuring consistent cardiomyocyte isolation across a wide range of tissue sizes.^9^^,^^13^, ^14^, ^15^

Following isolation, extracellular Ca^2+^ concentration was gradually raised to 2 mmol/L. For downstream experiments, exclusively elongated, non-granulated cardiomyocytes exhibiting cross-striations were chosen.^16^

### Murine cardiomyocytes isolation and in vitro AF simulation

Murine ventricular cardiomyocytes were isolated as previously described.^17^ Ca^2+^ was reintroduced gradually by stepwise increasing the extracellular Ca^2+^ concentration from 0.1 mmol/L to 1.4 mmol/L. Isolated cells were then cultured in 6-well plates under a final Ca^2+^ concentration of 2 mmol/L. To simulate AF in vitro, electrical field stimulation was applied using a culture stimulation system (C-Pace EM, IonOptix Corp) over a 24-hour period. Control cells were paced regularly at a constant frequency of 60 beats/min. In contrast, AF simulation was achieved using irregular pacing at the same average rate (60 beats/min), but with a beat-to-beat variability of 40%, mimicking normofrequent AF as described before.^9^^,^^12^ The beat-to-beat variability applied in our AF simulation was chosen to reflect clinically observed patterns of ventricular irregularity in patients with AF.^18^^,^^19^ Although murine heart rates are markedly higher in vivo, isolated cardiomyocytes are unable to tolerate the high pacing frequencies observed in mice over prolonged periods and the necessary stimulation voltage and pulse duration would further compromise cellular physiology.^12^ AF simulation was conducted for 24 hours because long-term in vitro culture of adult ventricular cardiomyocytes can lead to electrophysiological and structural deterioration of isolated cells.^20^ The stimulation pulse amplitude was set between 10% and 20% above the threshold to ensure effective capture of the cells. Cell viability and the effectiveness of electrical stimulation were regularly assessed throughout the experiment using light microscopy.

### Recording of spontaneous afterdepolarizations

Human and murine cardiomyocytes were incubated on laminin-coated measurement chambers with Tyrode's solution containing 2 mmol/L Ca^2+^ concentration for a 15-minute period before initiating measurements. To enhance the incidence of arrhythmogenic afterdepolarizations through beta-adrenergic stimulation, the Tyrode's solution for murine cardiomyocytes included 100 nmol/L isoproterenol.^21^ Microelectrodes (2 to 4 MΩ) were filled with an internal solution containing: K-aspartate 122 mmol/L, KCl 8 mmol/L, NaCl 10 mmol/L, MgCl2 1 mmol/L, HEPES 10 mmol/L, Mg-ATP 5 mmol/L, and LiGTP 0.3 (pH 7.2, KOH).

The ruptured-patch whole-cell current-clamp technique was used to measure action potentials which were continually elicited using current pulses (0.75-1 nA, 2-6 ms) at room temperature. Access resistance typically remained below 10 MΩ post-rupture. Fast capacitance was compensated in cell-attached configuration. Membrane capacitance and series resistance were compensated after patch rupture. Signals were filtered using 2.9- and 10-kHz Bessel filters and acquired with an EPC10 amplifier (HEKA Elektronik). For the recording of spontaneous afterdepolarizations, stimulation cycle of 30 beats/min was used, along with a stimulation protocol in which a triple 60 beats/min stimulation period was interrupted firstly by a 10-second pause and secondly by a 30-second pause and incidence of delayed afterdepolarizations (DADs) was counted.^22^

To assess the effect of NCX inhibition on the incidence of DADs, cardiomyocytes were superfused with Tyrode’s solution either in the absence or presence of the selective sodium-calcium exchanger (NCX) inhibitor ORM-10962 (1 μmol/L). Cells exposed to ORM-10962 were compared with cardiomyocytes recorded under identical conditions without NCX inhibitor application. Data analysis was performed using LabChart 8 software (ADInstruments).^22^^,^^23^

### Measurement of NCX current

Whole-cell patch-clamp recordings were performed in voltage-clamp mode at room temperature to measure NCX currents. Patch pipettes (2-3 MΩ) were filled with an internal solution containing: 10 mmol/L NaCl, 40 mmol/L Cs-glutamate, 90 mmol/L CsCl, 0.36 mmol/L CaCl_2_, 1 mmol/L EGTA (free intracellular Ca^2+^ concentration adjusted to 100 nmol/L), 5 mmol/L MgATP, 10 mmol/L tetraethylammonium chloride (TEA-Cl), and 10 mmol/L HEPES (pH adjusted to 7.2 with CsOH). The external bath solution contained: 130 mmol/L NaCl, 5 mmol/L CsCl, 10 mmol/L TEA-Cl, 1 mmol/L MgCl_2_, 10 mmol/L glucose, 10 mmol/L HEPES, 1.8 mmol/L CaCl_2_, and 0.02 mmol/L nifedipine (pH adjusted to 7.4 with CsOH). Cesium and TEA were used to suppress potassium currents, and nifedipine was included to inhibit L-type Ca^2+^ channels. Access resistance was typically <7 MΩ, and recordings were initiated 3 minutes after membrane rupture to allow equilibration of the intracellular solution. NCX currents were elicited using a voltage-ramp protocol from +80 mV to −120 mV (duration 4 seconds, ramp speed 50 mV/s) from a holding potential of −40 mV. I_NCX_ was defined as the current sensitive to NiCl_2_ (5 mmol/L) and normalized to membrane capacitance to obtain current density (pA/pF).^24^

### Confocal Ca^2+^ Spark measurements

All Ca^2+^ imaging experiments were performed under dim red-light conditions to minimize photobleaching. The Ca^2+^-sensitive dye Fluo-4 AM (Molecular Probes) was prepared by dissolving one 50-μg vial in 44.4 μL anhydrous dimethyl sulfoxide. From this stock, 10 μL were mixed with 1 μL Pluronic F-127 by repeated pipetting, followed by dilution in 1 mL Tyrode’s solution to yield a final Fluo-4 AM concentration of 10 μmol/L. Isolated ventricular cardiomyocytes were plated onto laminin-coated chambers and allowed to settle. Cells were then incubated with the Fluo-4 AM loading solution (500 μL for human cardiomyocytes) for 15 minutes at room temperature. Following incubation, the dye solution was carefully removed, and cells were washed and Tyrode’s solution was added for an additional 15 minutes. Ca^2+^ spark recordings were performed using a laser-scanning confocal microscope (Zeiss) in line-scan mode.^25^ To ensure adequate sarcoplasmic reticulum Ca^2+^ loading, recordings were acquired at rest following continuous field stimulation at 1 Hz. To minimize observer bias, Ca^2+^ spark analysis was performed in a blinded manner. Raw image files were anonymized before analysis by renaming file identifiers. Quantitative analysis of Ca^2+^ sparks was performed using SparkMaster, a validated, automated analysis plugin for ImageJ, which identifies Ca^2+^ release events in line-scan images based on a statistically defined threshold relative to baseline fluorescence (F_0_), allowing objective detection of localized, transient increases in intracellular Ca^2+^.

### Western blots

In vitro–paced murine ventricular cardiomyocytes were used to assess protein expression and oxidative modifications. Protein denaturation was performed under specific conditions tailored to each target. Ryanodine receptor type 2 (RyR2), RyR2-pS2814, phospholamban (PLB), PLB-pT17, and sarcoplasmic/endoplasmic reticulum Ca^2+^–adenosine triphosphatase 2a (SERCA2a) were denatured at 37 °C for 30 minutes in the presence of 10% β-mercaptoethanol. Ca^2+^/calmodulin-dependent protein kinase IIδ (CaMKIIδ) and glyceraldehyde 3-phosphate dehydrogenase (GAPDH) were denatured at 95 °C for 5 minutes with 10% β-mercaptoethanol. For oxidized CaMKII (CaMKII-Ox), denaturation was conducted at 95 °C for 5 minutes in the absence of reducing agents to preserve oxidation-sensitive epitopes.

Proteins were separated on sodium dodecyl sulfate–polyacrylamide gels of varying acrylamide concentrations, optimized for the molecular weight of each target: 5% for RyR2; and RyR2-pS2814 (565 kDa); 8% for CaMKII, CaMKII-Ox, and SERCA2a and NCX (108 kDa); and 11% for PLB. Following electrophoresis, proteins were transferred to nitrocellulose membranes and blocked before incubation with the respective primary antibodies: anti-RyR2 (rabbit polyclonal, 1:1,000, Sigma), anti-RyR2-pS2814 (rabbit polyclonal, 1:1,000, Badrilla), anti-SERCA2a (mouse monoclonal, 1:20,000, Thermo Scientific), anti-PLB (mouse monoclonal, 1:20,000, Invitrogen), anti-PLB-pThr17 (rabbit polyclonal, 1:1,000, Badrilla), anti-CaMKIIδ (rabbit polyclonal, 1:10,000, Thermo Fisher), anti-NCX1 (mouse monoclonal, 1:1,000, Abcam), and anti-GAPDH (mouse monoclonal, 1:10,000, Sigma). Oxidation of CaMKII at the methionine residues at positions 281 and 282 (Met281/282) was studied using a rabbit polyclonal anti-oxidized-CaMKII antibody (1:1,000, GeneTex).

Membranes were incubated with primary antibodies overnight at 4 °C, followed by 1-hour incubation at room temperature with appropriate horseradish peroxidase–conjugated secondary antibodies: donkey anti-rabbit or sheep anti-mouse immunoglobulin (1:10,000, GE Healthcare). Chemiluminescent detection was performed using Immobilon Western chemiluminescent horseradish peroxidase substrate (Millipore) on a Bio-Rad ChemiDoc MP system. Densitometric analysis of band intensity was performed using ImageJ2 (Fiji distribution, GitHub).^9^ Western blot signals were quantified using paired samples (AF simulation vs control) obtained from the same mouse. Incomplete pairs were excluded from analysis.

### Statistical analysis

Clinical and experimental data are presented at the level of the individual subject (patient or mouse). Continuous variables are reported as mean ± SD or median (Q1-Q3), as appropriate. Categorical variables are presented as absolute numbers and percentages.

Normal distribution of continuous variables was assessed using the Shapiro–Wilk test where applicable. Comparisons between 2 independent groups with normally distributed data were performed using an unpaired 2-tailed Student’s *t*-test. Paired analyses were conducted using a paired 2-tailed Student’s *t*-test. In cases of non-normally distributed data, the nonparametric Mann–Whitney U test was applied. Categorical variables were compared using the chi square test or Fisher exact test, as appropriate. These statistical analyses and data visualization were performed using GraphPad Prism version 10.0 (GraphPad Software).

Univariate linear regression analyses were performed to assess associations between the outcome variable DADs/min and clinically relevant covariates. The following variables were evaluated in univariate analyses: age (years), sex, body mass index (BMI, kg/m^2^), glomerular filtration rate (GFR, mL/min), glycated hemoglobin (HbA_1c_, %), left ventricular ejection fraction (LVEF, %), B-type natriuretic peptide (BNP, pg/mL), aortic valve mean gradient (mm Hg), and the presence of AF (yes vs no).

A multivariate linear regression model was constructed to evaluate the independent association between AF and DADs/min. Given the limited sample size, the number of covariates included in the multivariate model was restricted to avoid overfitting. The final multivariate model included AF (yes vs no), BMI, age, sex, GFR, HbA_1c_, LVEF, and BNP. The aortic valve mean gradient was analyzed in univariate regression only.

Only variables with data linked to DADs/min available in more than 20 subjects (n > 20) were included in the regression analyses. Regression coefficients (B) with corresponding 95% CIs are reported for all regression analyses. A 2-sided *P* < 0.05 was considered statistically significant. All regression analyses were performed using Stata (StataCorp).^26^

## Results

### Patient characteristics

This study investigated LV myocardium from 25 patients with SR and 16 patients with persistent or permanent AF. Patient characteristics are summarized in Table 1. Overall, baseline characteristics and comorbidities were comparable between groups. There was no significant difference in age between patients with SR and AF (66.6 ± 8.2 vs 69.2 ± 15.6 years), nor in BMI or renal function. Heart rate did not differ significantly between groups (SR: 65.0 [Q1-Q3: 64.0-71.0] beats/min; n = 15 vs AF: 71.5 [Q1-Q3: 65.0-83.8] beats/min; n = 10), allowing assessment of AF-associated effects largely independent of tachycardia.

LVEF was preserved and comparable in both groups (SR: 59.0% [Q1-Q3: 54.5%-60.0%]; n = 25 vs AF: 60% [Q1-Q3: 50.0%-60.0%]; n = 15). Severity of AS, assessed by aortic valve area and mean transvalvular gradient, as well as indices of concentric remodeling such as interventricular septal thickness, did not differ significantly between SR and AF patients. In contrast, left ventricular end-diastolic diameter was significantly larger in the AF group (SR: 47.1 ± 6.2 mm vs AF: 54.9 ± 8.5 mm; *P* = 0.007). Medication use, including β-blockers, renin–angiotensin system inhibitors, diuretics, and statins, was not statistically different between groups.

To identify clinical determinants of DAD frequency, univariate and multivariate linear regression analyses were performed; however, these can be limited by the small patient number on our study (Supplemental Table 1). In univariate analyses, the presence of AF was significantly associated with an increased frequency of DADs (B = 7.33; 95% CI: 1.33-13.32; *P* = 0.020). In contrast, no significant associations with DAD frequency were observed for BMI, age, male sex, GFR, LVEF, BNP (pg/mL), or aortic valve mean gradient. In multivariate analysis (n = 22) adjusting for AF, BMI, age, sex, renal function, glycemic control, LVEF, and BNP, AF remained independently associated with DAD frequency (B = 8.35; 95% CI: 0.84-15.86; *P* = 0.032). None of the other covariates, including BMI, age, male sex, GFR, HbA_1c_, LVEF, or BNP, showed independent associations with DAD frequency.

### AF increases the incidence of proarrhythmic triggers in human ventricular cardiomyocytes

**Figure 1:**
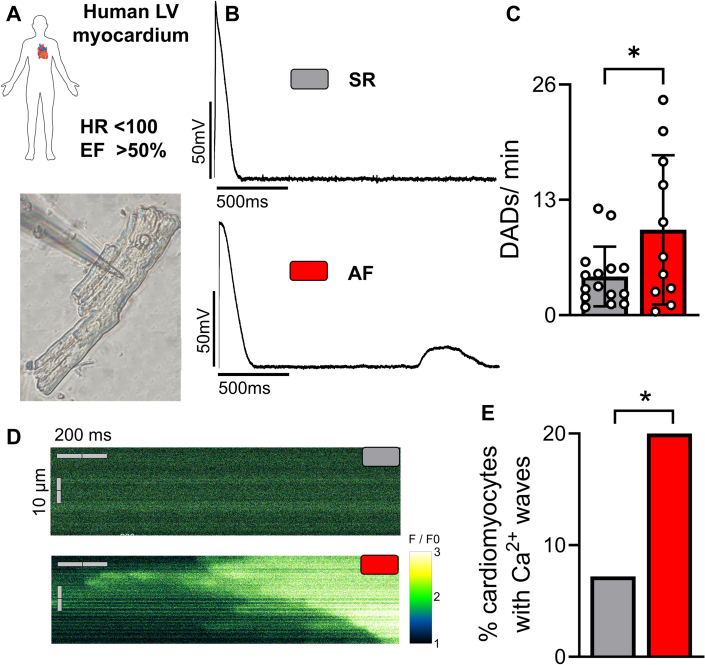
AF Increases Cellular Proarrhythmic Mechanisms in Human LV Cardiomyocytes (A) Patch pipette (shadow) after successful seal and rupture during whole cell current clamp recording of an isolated human ventricular cardiomyocyte. (B) Representative original recordings of stimulated action potentials of human left ventricular (LV) cardiomyocytes from patients with sinus rhythm (SR) or atrial fibrillation (AF) showing delayed afterdepolarization (DAD) in AF. (C) Mean values of DAD frequency (expressed in DADs/min) of human LV cardiomyocytes from patients with SR (n = 15; 59 cardiomyocytes) compared with AF (44 cardiomyocytes). Values from multiple cardiomyocytes of the same patient were averaged before statistical analysis. (D) Representative original recordings of Ca^2+^ waves comparing cardiomyocytes from patients with SR or AF. (E) Percentage of human LV cardiomyocytes from patients with SR (n = 134 cardiomyocytes; 10 patients) compared with AF (60 cardiomyocytes; 5 patients) showing Ca^2+^ waves. Data are presented as bar graphs showing mean ± SD. Asterisks indicate statistical significance: ∗*P* < 0.05. Groups were compared using unpaired Student’s *t*-test (for C) or chi square test (for E). EF = ejection fraction; HR = heart rate.

We investigated whether AF is associated with ventricular arrhythmogenesis by isolating human LV cardiomyocytes for patch clamp studies (Figure 1A). DADs (Figure 1B), which are irregular cellular depolarizations that can evoke spontaneous action potentials and are well-established cellular arrhythmic triggers promoting ventricular arrhythmias,^27^, ^28^, ^29^ were found to occur at a significantly increased frequency in cardiomyocytes from AF patients (n = 11; 44 cardiomyocytes) compared to SR patients (n = 15; 59 cardiomyocytes) (Figure 1C). Our electrophysiological findings were corroborated by confocal Ca^2+^ imaging, which revealed a higher incidence of spontaneous Ca^2+^ waves — major irregular diastolic Ca^2+^ release events from the sarcoplasmic reticulum (Figure 1D). Notably, arrhythmic events were observed in 20% of cardiomyocytes from AF patients (60 cardiomyocytes; n = 5), whereas only 7.2% of cells in the SR group (134 cardiomyocytes; 10 patients, (Figure 1E) exhibited such abnormalities. As diastolic Ca^2+^ can induce a depolarizing inward current via NCX,^30^ the increase in Ca^2+^ waves may underlie the cellular DADs observed in AF cardiomyocytes. These data from human LV myocardium show that patients with AF exhibit ventricular proarrhythmic triggers, which may contribute to the increased risk of ventricular arrhythmias and SCD.

### In vitro simulation of AF induces proarrhythmic mechanisms in murine ventricular cardiomyocytes

We sought to build upon these findings suggesting an impact of AF on LV cardiomyocyte arrhythmogenesis independent of heart rate. To mitigate the impact of confounding factors in patients such as medication and comorbidities, we studied murine ventricular myocytes subjected to irregular pacing mimicking conducted AF. Importantly, we chose an in vitro system for AF simulation because most in vivo animal models do not develop spontaneous persistent/permanent AF.^31^ In vivo pacing in animals is insufficient to simulate the irregular ventricular excitation via normal atrioventricular node conduction. In fact, in vivo ventricular [pacing] per se may impair ventricular function due to ventricular dyssynchrony.^32^^,^^33^ We used murine adult ventricular cardiomyocytes in this study because immature cardiomyocyte models such as induced pluripotent stem cell cardiomyocytes are limited for investigating arrhythmogenesis due to intrinsic depolarizations and spontaneous beating.

**Figure 2:**
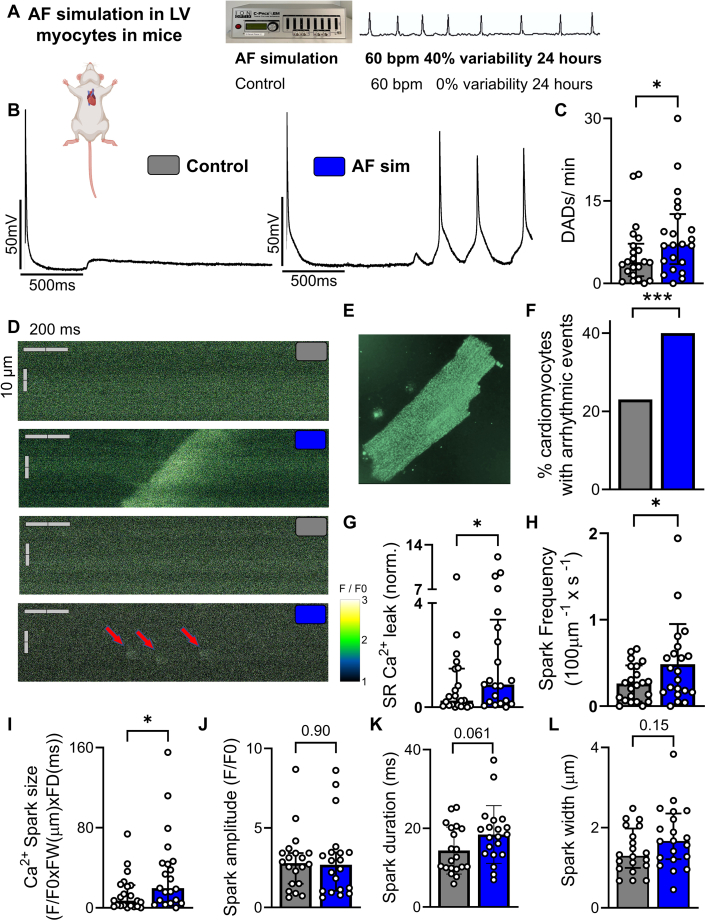
AF Simulation Promotes Ventricular Arrhythmogenesis in Mice (A) Schematic representation of the AF simulation protocol and exemplary electrocardiogram (ECG) trace from a patient with AF. Normofrequent conducted AF was simulated by irregular pacing at 60 beats/min with a beat-to-beat variability of 40%. (B) Representative original recordings of stimulated action potentials and (C) mean values of DAD frequency of murine LV cardiomyocytes subjected to AF simulation (n = 22 mice; 46 cardiomyocytes) compared with control pacing (22; 51 cardiomyocytes). (D) Original recordings from murine ventricular cardiomyocytes by confocal laser fluorescence microscopy in line-scan mode after (E) Ca^2+^ staining with Fluo-4. (F) Percentage of cardiomyocytes showing arrhythmic events comparing control (n = 172 cardiomyocytes; 22 mice) to AF simulation (175 cardiomyocytes; 21 mice). (G) Quantification of Ca^2+^ spark frequency and (H) normalized diastolic sarcoplasmic reticulum Ca^2+^ leak (control: 22 mice, 172 cardiomyocytes; AF simulation: 21 mice, 175 cardiomyocytes). (I-L) Ca^2+^ sparks size, amplitude, duration and width. Ca^2+^ spark size was calculated as F/F_0_ × FW × FD. Data are presented as mean ± SD for parametric variables and median with Q1-Q3 for nonparametric variables. Asterisks indicate statistical significance: ∗*P* < 0.05, ∗∗*P* < 0.01, and ∗∗∗*P* < 0.001. Values from multiple cardiomyocytes of the same mouse were averaged before statistical analysis, unless otherwise specified (see F). Groups were compared using unpaired Student’s *t*-test (H and K) and Mann-Whitney U test (unpaired; for C, G, I, J, and L) or chi square test (for F). F/F_0_ = peak fluorescence normalized to baseline fluorescence; FD = full duration; FW = full width; other abbreviations as in Figure 1.

AF was simulated in vitro by using a beat-to-beat variability of 40% at an average stimulation frequency of 60 beats/min (Figure 2A) to mimic human normofrequent AF. We did not observe harmful effects of electric pacing for 24 hours on cell viability (Supplemental Figure 1). AF simulation increased the incidence of DADs in ventricular cardiomyocytes (Figure 2B) compared to regular pacing at the same rate, with AF simulated myocytes (22 mice; 46 cardiomyocytes) exhibiting an increase in DADs by 65% compared to regular pacing conditions (22 mice; 51 cardiomyocytes) (Figure 2C).

We next performed confocal Ca^2+^ imaging (Figures 2D and 2E) and reproduced the findings observed in human LV cardiomyocytes: The fraction of ventricular cardiomyocytes exhibiting proarrhythmic Ca^2+^ events increased markedly from 22.7% in the control group (172 cardiomyocytes; 22 mice) to 40.0% after AF simulation (175 cardiomyocytes; 21 mice) (Figure 2F). Moreover, AF simulation resulted in significantly elevated diastolic Ca^2+^ leak and Ca^2+^ spark frequency, indicative of disturbed Ca^2+^ handling (control: 22 mice; 172 cardiomyocytes vs AF simulation: 21 mice; 175 cardiomyocytes) (Figures 2G and 2H). In addition, overall Ca^2+^ spark size was significantly increased in AF simulated cardiomyocytes without significant changes in individual Ca^2+^ spark parameters (Figures 2I to 2L). These results demonstrate the proarrhythmic effects of irregular pacing on ventricular cardiomyocytes that may explain increased ventricular arrhythmias in the setting of normofrequent human AF.

### Increased oxidative CaMKII activation in murine ventricular cardiomyocytes in response to AF simulation

**Figure 3:**
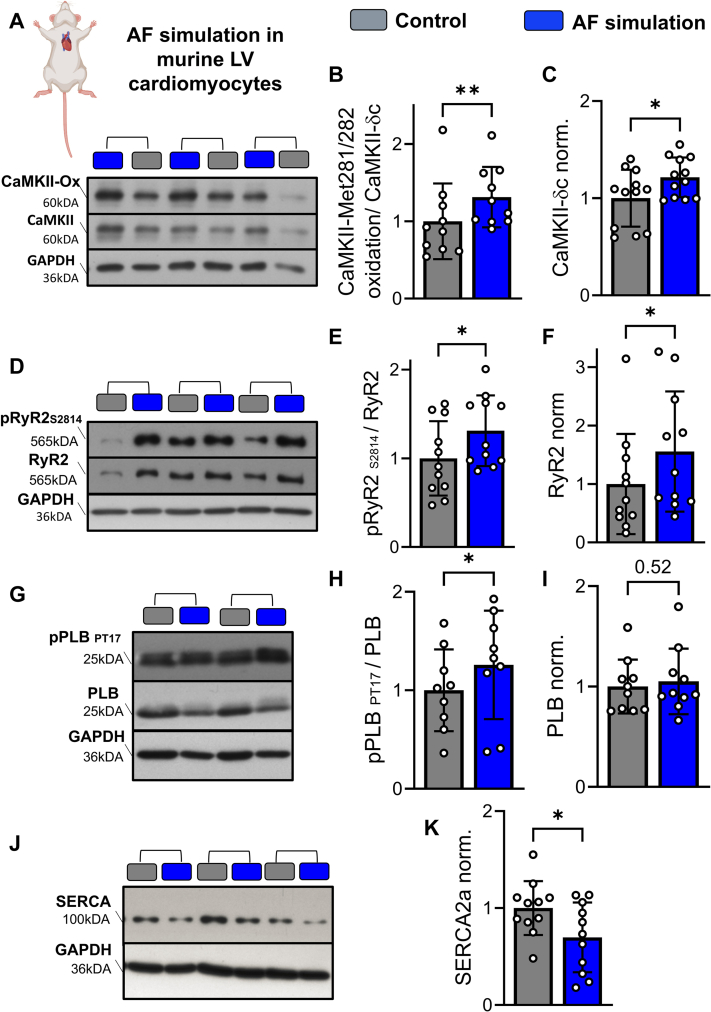
AF Simulation Increases CaMKII Oxidation and CaMKII-Dependent Phosphorylation of Ca^2+^-Handling Proteins (A) Original representative Western Blots of murine LV cardiomyocytes after 24-hour AF simulation showing oxidized Ca^2+^/calmodulin-dependent protein kinase II (CaMKII) at Met281/282 (CaMKII-ox), total CaMKII, and glyceraldehyde 3-phosphate dehydrogenase (GAPDH). (B) Quantification of CaMKII oxidation normalized to total CaMKII (CaMKII-ox/CaMKII).(C) Quantification of total CaMKII expression normalized to GAPDH (n = 10 to 12 mice). (D) Original representative Western Blots of phosphorylated ryanodine receptor type 2 (RyR2) at Ser2814 (p-RyR2-S2814), total RyR2, and GAPDH. (E) Quantification of p-RyR2-S2814 normalized to total RyR2. (F) Quantification of total RyR2 expression normalized to GAPDH (11 mice). (G) Original representative Western Blots of phosphorylated phospholamban at Thr17 (p-PLB-T17), total phospholamban (PLB), and GAPDH. (H) Quantification of p-PLB-T17 normalized to total PLB. (I) Quantification of total PLB expression normalized to GAPDH (9 to 10 mice). (J) Original representative Western Blots of sarcoplasmic/endoplasmic reticulum Ca^2+^–adenosine triphosphatase 2a (SERCA2a) and GAPDH. (K) Quantification of SERCA2a expression normalized to GAPDH (11 mice). Data are presented as bar plot with mean ± SD. Asterisks indicate statistical significance: ∗*P* < 0.05, ∗∗*P* < 0.01, ∗∗∗*P* < 0.001. Groups were compared using unpaired Student’s *t*-test. Abbreviations as in Figure 1.

To elucidate the molecular mechanisms underlying the observed alterations in Ca^2+^ handling, we performed Western blot analyses of key Ca^2+^-regulatory proteins. AF simulation for 24 hours resulted in a significant upregulation of total CaMKII expression accompanied by a 31.2% increase CaMKII oxidation at the regulatory oxidation site Met281/282 (10 to 12 mice each) (Figures 3A to 3C).

As CaMKII oxidation is known to result in increased CaMKII activity^34^ and thus, to enhance phosphorylation of Ca^2+^ handling proteins,^35^ we next assessed the expression and phosphorylation status of the RyR2. AF simulation induced a significant increase in RyR2 expression together with hyperphosphorylation at the CaMKII-dependent Ser2814 site (n = 11) (Figures 3D to 3F), consistent with enhanced RyR2 open probability and proarrhythmogenic diastolic Ca^2+^ release from the sarcoplasmic reticulum.^36^ We further extended these analyses to additional CaMKII downstream targets. Although total PLB expression remained unchanged, phosphorylation of PLB at the CaMKII-specific Thr17 residue was significantly increased following AF simulation (n = 9 to 10) (Figures 3G to 3I), consistent with increased CaMKII activation. SERCA2a protein expression was significantly reduced in AF-simulated cardiomyocytes (n = 11) (Figures 3J to 3K), which may further contribute to increased diastolic Ca^2+^ levels. Collectively, these findings show that AF simulation induces oxidative CaMKII activation in ventricular cardiomyocytes, leading to hyperphosphorylation of key downstream targets such as RyR2, which can explain the proarrhythmic alterations in Ca^2+^ cycling.

### AF simulation enhances NCX expression and function

**Figure 4:**
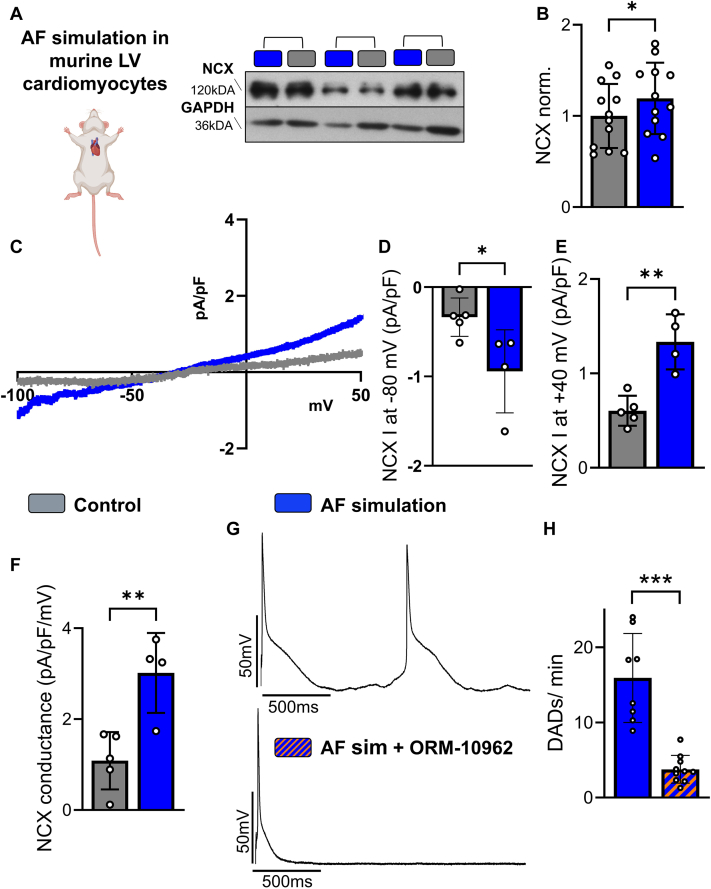
AF Simulation Results in Enhanced NCX Expression and Activation (A and B) Original representative Western blots and corresponding quantitative analysis of sodium-calcium exchanger (NCX) expression in murine LV cardiomyocytes after 24 hours of AF simulation (n = 12 mice). GAPDH served as loading control. (C) Representative original recordings of NCX current and quantification of NCX current density at −80 mV (D) and +40 mV (E), as well as NCX conductance (F) of murine LV cardiomyocytes after 24 hours of AF simulation (n = 4 to 5 mice; 6 cardiomyocytes each). (G) Representative original action potential recordings from murine LV cardiomyocytes after 24 hours of AF simulation, obtained in the absence and presence of the NCX inhibitor ORM-10962. (H) Mean values of DAD frequency in murine LV cardiomyocytes subjected to AF simulation (8 mice; 16 cardiomyocytes) and after treatment with ORM-10962 (10 mice; 18 cardiomyocytes). Data are presented as bar plot with mean ± SD. Asterisks indicate statistical significance: ∗*P* < 0.05, ∗∗*P* < 0.01, ∗∗∗*P* < 0.001. Values from multiple cardiomyocytes of the same mouse were averaged before statistical analysis. Statistical comparisons were performed using unpaired Student’s *t*-test. Abbreviations as in Figure 1, Figure 2, Figure 3.

NCX is the main contributor for generating a depolarizing inward current in response to (spatiotemporally) increased Ca^2+^ levels, for example, during diastolic Ca^2+^ waves.^37^ Therefore, we assessed NCX expression and function in murine LV cardiomyocytes following 24 hours of AF simulation. Western blot analysis revealed a significant 19.3% increase in NCX protein expression in murine ventricular cardiomyocytes after AF simulation compared with control conditions (n = 12) (Figures 4A and 4B). To determine whether AF simulation also resulted in increased NCX function, NCX currents were measured using whole-cell voltage-clamp recordings (Figure 4C). Quantitative analysis showed a significant increase in NCX current density at −80 mV as well as at +40 mV in cardiomyocytes subjected to AF simulation (4 mice; 6 cardiomyocytes) compared with controls (5 mice; 6 cardiomyocytes) (Figures 4D and 4E). In addition, the slope of the NCX current–voltage relationship, reflecting NCX conductance, was significantly increased after AF simulation, indicating enhanced overall exchanger activity (Figure 4F). Next, we tested whether NCX inhibition can prevent cellular arrhythmias. In whole-cell current-clamp recordings, murine ventricular cardiomyocytes subjected to AF simulation (8 mice; 16 cardiomyocytes) exhibited a high incidence of DADs. In contrast, cardiomyocytes treated with the selective NCX inhibitor ORM-10962 (1 μmol/L; n = 10; 18 cardiomyocytes) showed a markedly reduced DAD incidence, corresponding to a 76.4% reduction (Figures 4G and 4H). Together, these findings identify enhanced NCX activity as a key contributor to AF-induced ventricular arrhythmogenesis, mechanistically linking increased diastolic Ca^2+^ release to the occurrence of afterdepolarizations.

### Genetic ablation of CaMKII oxidation protects against AF-induced arrhythmogenesis

**Figure 5:**
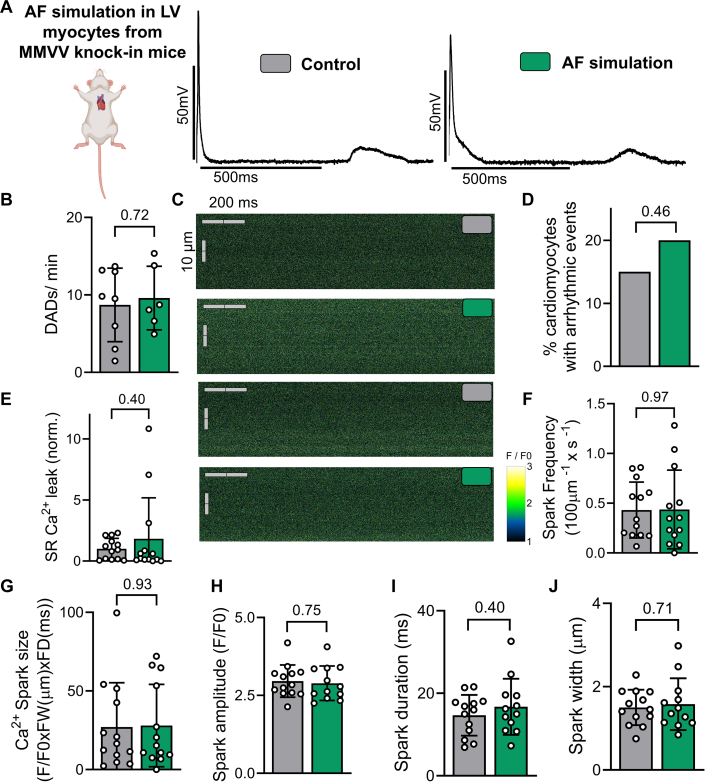
AF Simulation Does Not Induce Arrhythmogenesis in LV Cardiomyocytes From MMVV Knock-in Mice LV cardiomyocytes from knock-in mice lacking regulatory CaMKII oxidation sites Met281/282 (MMVV) after 24-hour in vitro AF simulation and (A) representative original recordings of action potentials. (B) Mean values of DAD frequency in LV cardiomyocytes from MMVV mice subjected to AF simulation (6 mice; 22 cardiomyocytes) compared with control (redundant, 8 mice; 27 cardiomyocytes). (C) Original recordings from confocal laser fluorescence microscopy in line-scan mode. (D) Percentage of cardiomyocytes showing arrhythmic events comparing AF simulation (110 cardiomyocytes; 13 mice) to control (102 cardiomyocytes; 13 mice). (E) Normalized diastolic sarcoplasmic reticulum Ca^2+^ leak and (F) Ca^2+^ spark frequency in LV cardiomyocytes from MMVV mice upon AF simulation (13 mice; 110 cardiomyocytes) vs control (13 mice; 102 cardiomyocytes). (G to J) Ca^2+^ sparks size, amplitude, duration and width. Ca^2+^ spark size was calculated as F/F_0_ × FW × FD. Data are presented as bar graphs showing mean ± SD. Values from multiple cardiomyocytes of the same mouse were averaged before statistical analysis, unless otherwise specified (see D). Groups were compared using unpaired Student’s *t*-test or chi square test (for D). Abbreviations as in Figure 1, Figure 2, Figure 3.

To test the causal relevance of oxidative CaMKII activation for arrhythmogenesis in ventricular cardiomyocytes upon AF, we used knock-in mice lacking regulatory CaMKII oxidation sites Met281/282 (MMVV).^38^ In these mice, the oxidation-prone methionine residues of CaMKII are substituted with valine, rendering the kinase resistant to oxidative modification. In contrast to C57BL6 mouse cardiomyocytes, AF simulation in MMVV ventricular cardiomyocytes did not impact the incidence of DADs (AF simulation: 6 mice; 22 cardiomyocytes vs control: 8 mice; 27 cardiomyocytes) (Figures 5A and 5B).

Confocal imaging to assay spontaneous Ca^2+^ release further confirmed the protective effect of ablating CaMKII oxidation. The percentage of cardiomyocytes displaying arrhythmic Ca^2+^ events (Figure 5C) was unchanged between control (15%, 102 cardiomyocytes; 13 mice) and AF simulation (20%, 110 cardiomyocytes; 13 mice) (Figure 5D). Similarly, neither the frequency of Ca^2+^ sparks nor the diastolic SR Ca^2+^ leak was elevated under AF conditions (13 mice; 110 cardiomyocytes) compared to control pacing (13 mice; 102 cardiomyocytes) in MMVV cardiomyocytes (Figures 5E and 5F). In addition, a detailed analysis of Ca^2+^ spark morphology revealed no significant differences in spark size, amplitude, duration, and width between control and AF simulated MMVV cells (Figures 5G to 5J). These findings underscore the involvement of oxidative CaMKII activation in AF-induced ventricular proarrhythmia and present CaMKII oxidation as a potential therapeutic target.

## Discussion

Ventricular arrhythmias are a main cause of mortality in patients with AF, with clinical studies identifying SCD as the leading cause of death in this population.^2^^,^^39^ Recent data have shown that AF strongly correlates with ventricular arrhythmias and cardiac arrest in patients with heart failure with preserved ejection fraction.^40^ The association between AF and SCD remains significant even after adjusting for shared cardiovascular risk factors.^41^ Accordingly, AF has also been identified as an independent risk factor for ventricular arrhythmias.^4^^,^^42^^,^^43^ A prospective study has shown that persistent AF was independently associated with a higher incidence in implantable cardioverter-defibrillator therapies for ventricular arrhythmias. This effect was potentially driven by irregular ventricular activation, particularly short-long-short sequences, rather than baseline heart rate.^44^ Despite this association, the mechanisms through which AF contributes to potentially life-threatening ventricular arrhythmias have remained poorly understood. In our translational study, we investigated that normofrequent AF ignites an arrhythmogenic cascade in ventricular cardiomyocytes by oxidative CaMKII activation and Ca^2+^ disturbances resulting in spontaneous depolarizations. This spontaneous activity can result in premature ventricular complexes/beats that can serve as spontaneous focal triggers or contribute to conduction/repolarization heterogeneity that are necessary for re-entry.^45^, ^46^, ^47^, ^48^

Although our patient cohort had a preserved ejection fraction, our observations in human myocardium may be influenced by clinical comorbidities and medication. Using our AF simulation model in LV cardiomyocytes from wild-type mice, we confirmed that the irregular ventricular response in AF, independent of tachycardia and in the absence of comorbidities or medication, is sufficient to induce ventricular arrhythmogenesis. We found that the proarrhythmic cascade in ventricular cardiomyocytes upon AF was promoted by oxidative CaMKII activation resulting in hyperphosphorylation of Ca^2+^-handling proteins. However, other CaMKII-dependent and/or independent pathways may also be affected by AF and should be considered in future research. Although previous work has described excitation-contraction coupling and contractility defects in AF ventricles,^9^ this study provides first translational and mechanistic evidence that AF promotes ventricular arrhythmogenesis. Key advances of this study include the identification of increased arrhythmogenic triggers in the ventricles of patients with AF, mechanistic insights into NCX-dependent arrhythmogenesis, and causal genetic validation in MMVV mice.

Our findings may provide a new mechanistic rationale on how rhythm control therapy may benefit patients with AF. Rhythm control strategies have been shown to significantly improve cardiovascular outcomes in patients with AF, as evidenced by several landmark clinical trials.^5^, ^6^, ^7^ Notably, the EAST-AFNET 4 trial has shown that early rhythm control in patients with recent-onset AF leads to a reduction in cardiovascular events compared to rate control.^8^ Similarly, the CASTLE-AF (Catheter Ablation versus Standard Conventional Therapy in Patients with Left Ventricular Dysfunction and Atrial Fibrillation) trial highlighted that catheter ablation not only improves symptoms but also reduces all-cause mortality in patients with heart failure with reduced ejection fraction and concomitant AF.^5^ The APAF-CRT (Ablate and Pace for Atrial Fibrillation - Cardiac Resynchronization Therapy) trial suggested the possible role for regularized ventricular pacing in reducing morbidity and mortality.^49^ Given that ventricular arrhythmias are a main driver of mortality in patients with AF,^2^^,^^3^ our data further underscore the harmful effects of arrhythmic ventricular activation in AF.

Targeting CaMKII oxidation may offer a potential therapeutic avenue, especially for AF patients who are ineligible for catheter ablation or in whom restoration of SR is not achievable. Previous studies have shown that reducing reactive oxygen species improves Ca^2+^ handling.^9^ Additionally, CaMKII inhibition has shown antiarrhythmic effects in preclinical models.^50^ General CaMKII inhibitors often lack specificity and pose systemic side effects. In contrast, specific ablation of the CaMKII oxidation site using gene-editing technologies such as CRISPR-Cas9 could enable a localized, cardiac-specific therapeutic approach.^51^

## Conclusions

Our study supports the view that AF is not only a disorder of the atrium, but a condition that affects the entire heart. Ventricular arrhythmias are a leading cause of death in our society.^2^ The role of AF in promoting ventricular arrhythmogenesis has important clinical implications and warrants further investigations.

Perspectives **COMPETENCY IN MEDICAL KNOWLEDGE:** Clinical trials indicated improved mortality in patients with AF when SR is maintained. Although stroke and HF are clinically appreciated complications of AF, the impact of AF on ventricular arrhythmias and potentially SCD is less understood. Our study establishes that AF promotes ventricular arrhythmogenesis. Electrophysiological investigations of human and murine ventricular myocardium have revealed for the first time an increased cellular arrhythmic trigger in AF based on the arrhythmic excitation of ventricular cardiomyocytes. These mechanisms are well established drivers of ventricular arrhythmia.

**TRANSLATIONAL OUTLOOK:** Future studies are needed to characterize the proarrhythmic ventricular phenotype upon AF in vivo and to evaluate the effects of SR restoration on ventricular tachycardia and ventricular fibrillation. Our study highlights an underappreciated, but potentially prognosis-relevant complication of AF. Further work is needed to define potential implications on clinical management of AF patients.

## Funding Support and Author Disclosures

Dr Spangler has received grants from the German Society of Cardiology. Dr Bapat has received grants from NIH K08HL171874. Dr Maier has received grants from the Deutsche Forschungsgemeinschaft (DFG) grant SFB TR374, DFG grant MA 1982/11-1, and an EU grant (Horizon 2020) STRATIFY-HF ID 101080905. Dr Sossalla has received grants from the Deutsche Forschungsgemeinschaft (DFG) through the research grant SO 1223/4-1 and the F. Thyssen Foundation (Az 10.19.2.026MN). Dr Wachter has received grants from the Deutsche Forschungsgemeinschaft WA 3075/6-1/-2/-3. Dr Pabel has received grants from the Else-Kröner-Fresenius Stiftung, by the German Heart Foundation/German Foundation of Heart Research, by the University of Regensburg (ReForM A program), and by the Deutsche Forschungsgemeinschaft (DFG) Walter Benjamin Programme (#530157297); he is also employed by the Novartis Institutes for Biomedical Research.
