# Supplementary material for: Atrial Fibrillation Increases Proarrhythmic Mechanisms in the Ventricle
Source: JACC Basic Transl Sci. 2026 May 7;11(6):101558. doi: 10.1016/j.jacbts.2026.101558 (PMC13185986; doi:10.1016/j.jacbts.2026.101558)
Supplement: Supplemental Table 1 and Supplemental Figure 1 [file mmc1.docx]

**Supplemental Material**

| **N = 22** | **Univariate linear regression analyses** | |  | **Multivariate linear regression analysis R² = 0.51** | |  |
| --- | --- | --- | --- | --- | --- | --- |
| **Variable: DADs/min** | **B (95 % CI)** | **p-value** |  | **B (95 % CI)** | **p-value** | |
| AF (yes vs. no) | 7.33 (1.33 – 13.32) | 0.020 |  | 8.35 (0.84 – 15.86) | 0.032 | |
| BMI, kg/m² | 0.39 (−0.13-0.91) | 0.14 |  | 0.49 (-0.16 – 1.15) | 0.12 | |
| Age, y | −0.04 (−0.44-0.36) | 0.85 |  | -0.07 (-0.54 – 0.39) | 0.74 | |
| Male sex | −1.14 (−6.73-4.45) | 0.68 |  | 0.54 (-5.97 – 7.05) | 0.86 | |
| GFR, ml/min | 0.02 (−0.11-0.14) | 0.80 |  | 0.08 (-0.07 – 0.23) | 0.28 | |
| HbA1c, % | 0.97 (−1.23-3.17) | 0.37 |  | 0.50 (-2.01 – 3.00) | 0.67 | |
| LVEF, % | -0.01 (-0.31 - 0.30) | 0.96 |  | 0.03 (-0.29 – 0.38) | 0.79 | |
| BNP,  per 100 pg/ ml | 0.03 (-0.04 to 0.10) | 0.42 |  | -0.016 (-0.114 – 0.082) | 0.72 | |
| AV mean gradient, mmHg | -0.066 (-0.252, 0.121) | 0.47 |  |  |  | |

**Supplemental Table 1. Univariate and multivariate linear regression analyses of determinants of delayed afterdepolarization frequency.** Univariate and multivariate linear regression analyses were performed to identify clinical and echocardiographic predictors of delayed afterdepolarizations per minute (DADs/min) in a cohort of n = 22 subjects. Only variables with available data in more than 20 patients (n > 20) were included in the regression analyses. Variables evaluated in univariate analyses were atrial fibrillation (AF; yes vs. no), body mass index (BMI), age, sex, glomerular filtration rate (GFR), glycated hemoglobin (HbA1c), left ventricular ejection fraction (LVEF), B-type natriuretic peptide (BNP) and aortic valve (AV) mean gradient. The multivariate model included AF, BMI, age, sex, GFR, Hb1c, LVEF and BNP. In the multivariate analysis, AF and BMI were independently associated with increased DADs/min, whereas other covariates were not. Only variables with data linked to DADs/min available in more than 20 subjects (n > 20) were included in the regression analyses. Regression coefficients (B) are presented with 95% confidence intervals and corresponding p-values.


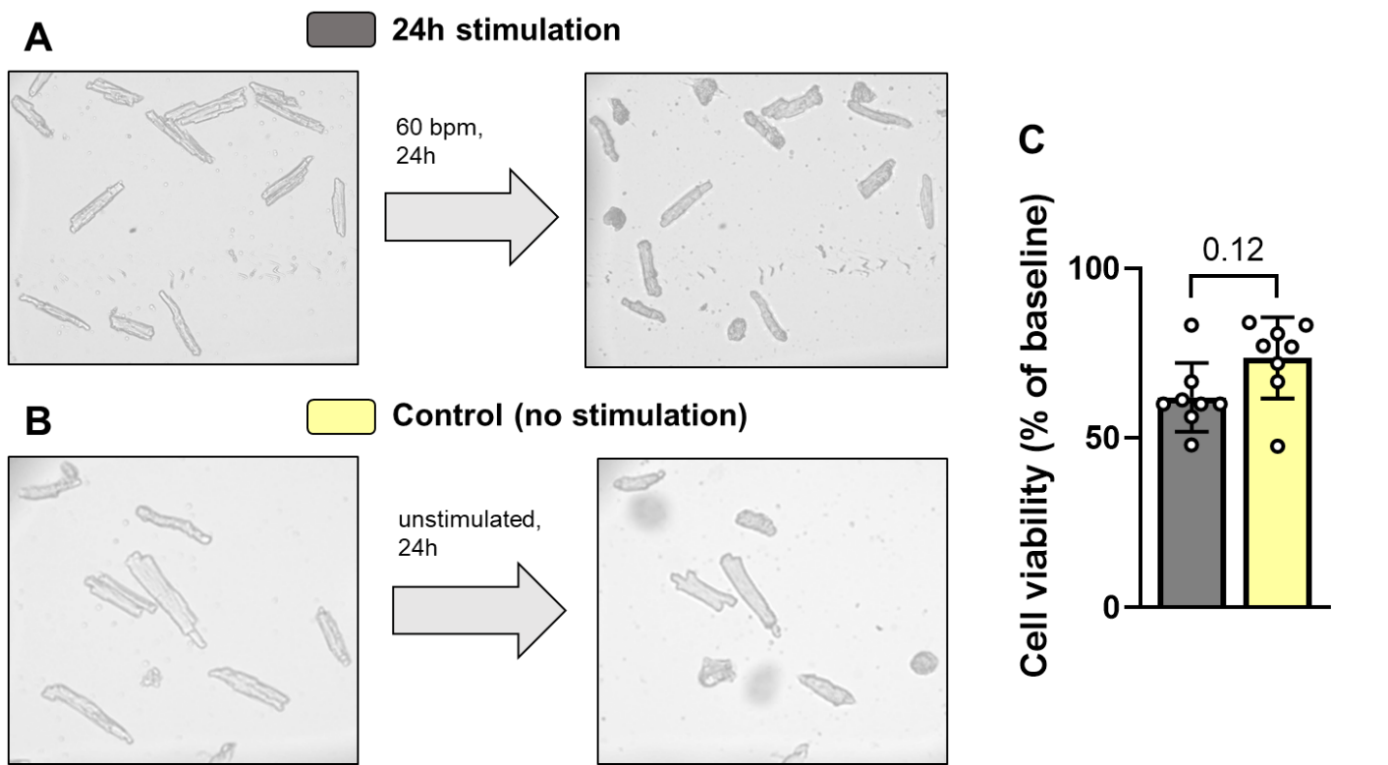


**Supplemental Figure 1: Cell viability after chronic pacing. (A)** Representative images of murine cardiomyocytes before pacing and after 24 h of electrical stimulation at a rate of 60 bpm. **(B)** Representative images of control cardiomyocytes maintained under identical culture conditions but without electrical stimulation for 24 h, shown before and after the corresponding time period. **(C)** Quantitative analysis of cell viability (% live cells with rod-shaped appearance, clear cross-striations, and absence of hypercontracture) after 24 h of culture with and without electrical stimulation. Data are presented as scatter plots with mean ± SD. Statistical comparison was performed using an unpaired t-test.
