# Supplementary material for: Atrial Fibrillation Increases Proarrhythmic Mechanisms in the Ventricle
Source: JACC Basic Transl Sci. 2026 May 7;11(6):101558. doi: 10.1016/j.jacbts.2026.101558 (PMC13185986; doi:10.1016/j.jacbts.2026.101558)
Supplement: Supplemental Material [file mmc2.pdf]

Original unedited full gel blots

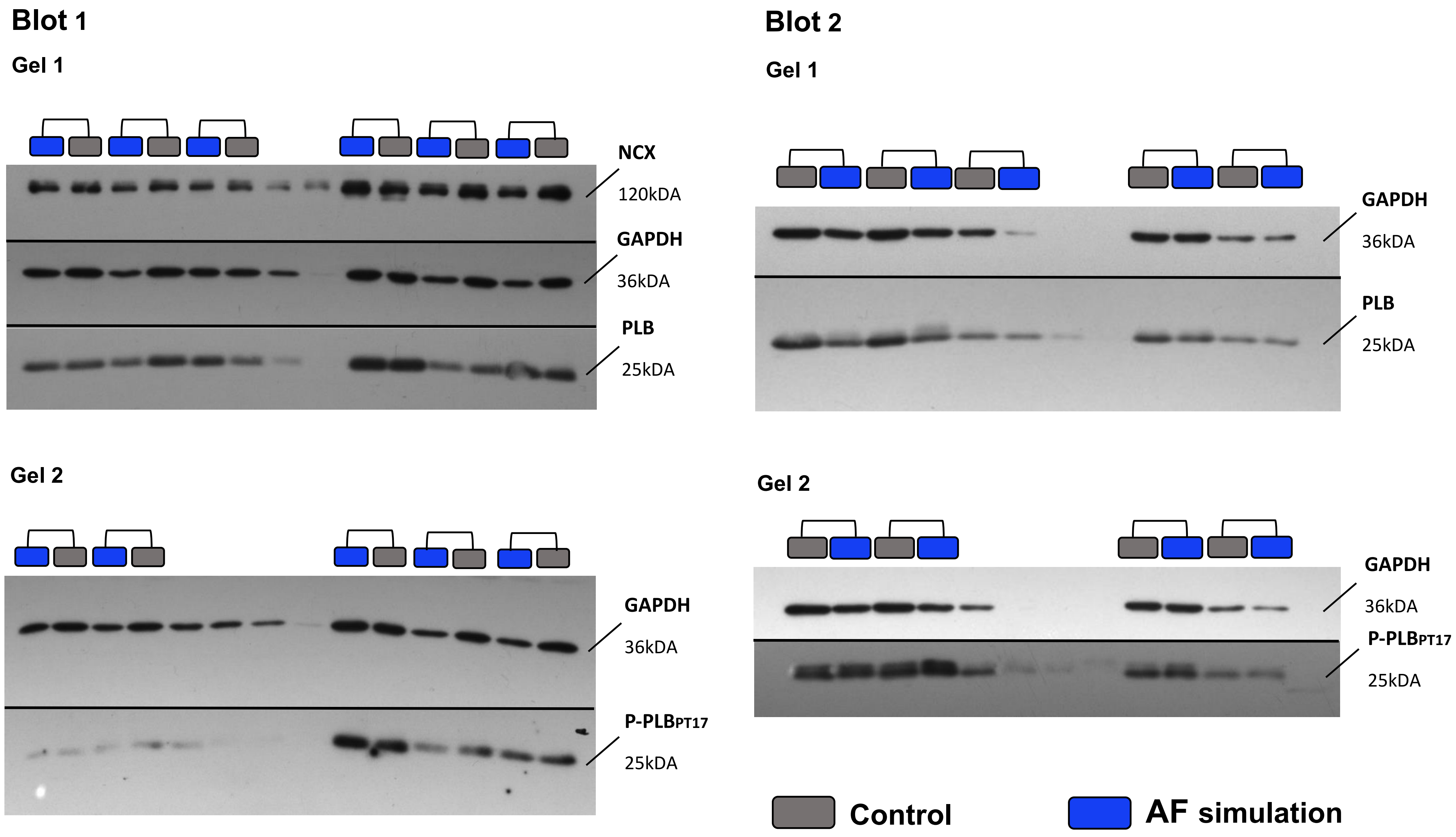
Blot 1
Gel 1
NCX
120kDA
GAPDH
36kDA
PLB
25kDA
Gel 2
GAPDH
36kDA
P-PLBPT17
25kDA
Blot 2
Gel 1
GAPDH
36kDA
PLB
25kDA
Gel 2
GAPDH
36kDA
P-PLBPT17
25kDA
Control
AF simulation

Blot 3

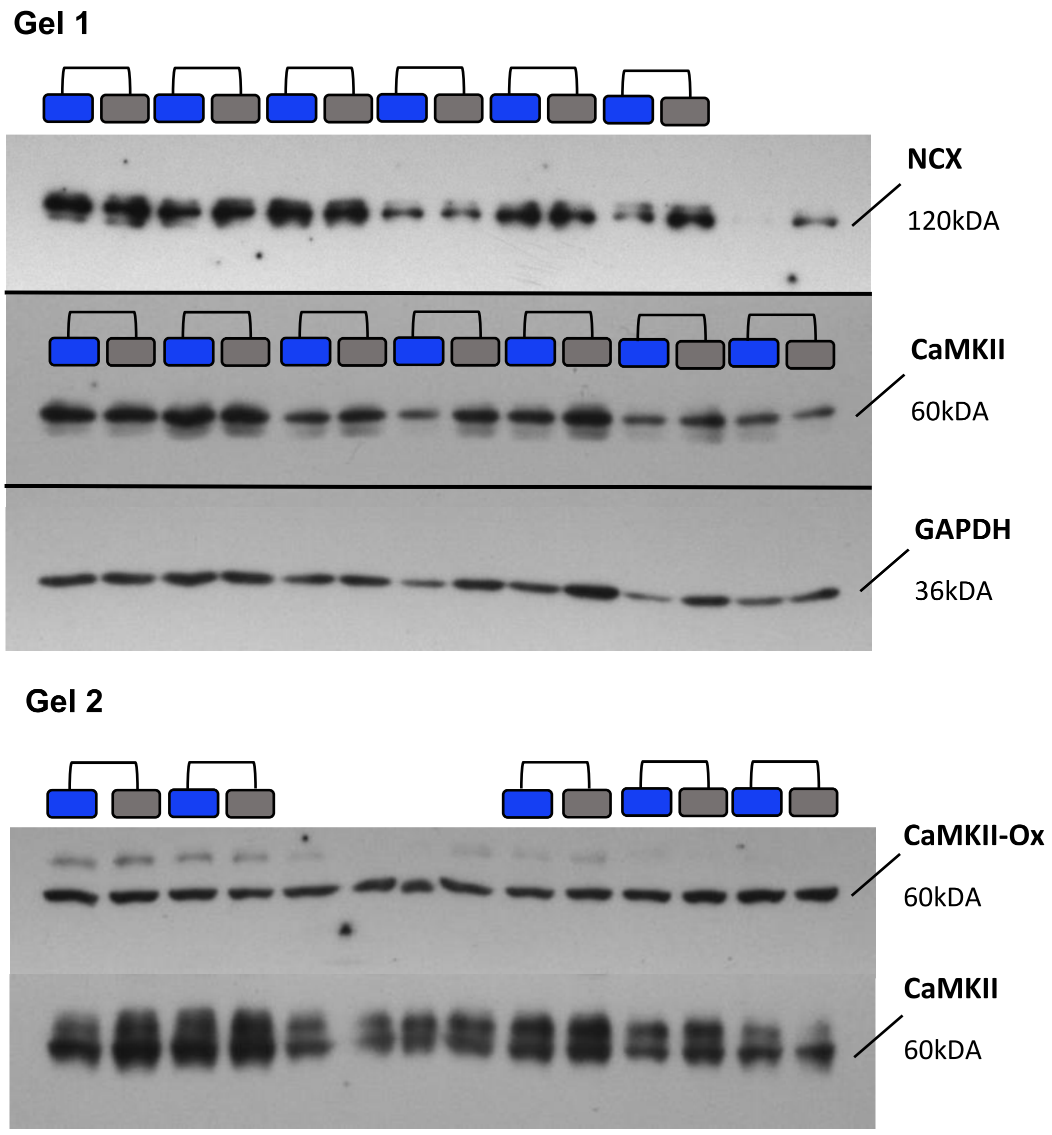
Gel 1
NCX
120kDA
CaMKII
60kDA
GAPDH
36kDA
Gel 2
CaMKII-Ox
60kDA
CaMKII
60kDA

Blot 4

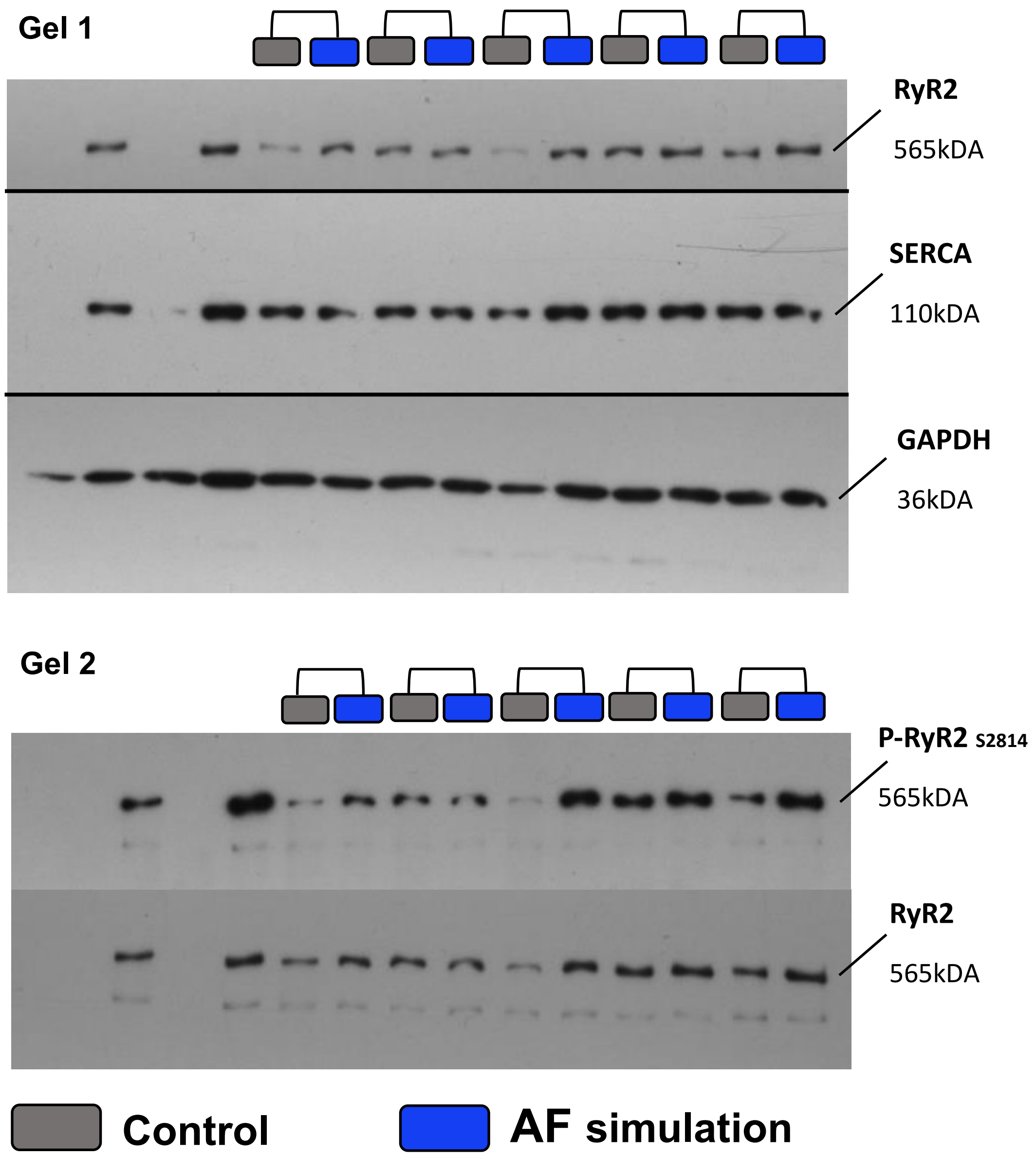
Gel 1
RyR2
565kDA
SERCA
110kDA
GAPDH
36kDA
Gel 2
P-RyR2 S2814
565kDA
RyR2
565kDA
Control
AF simulation

Blot 5

Gel 1

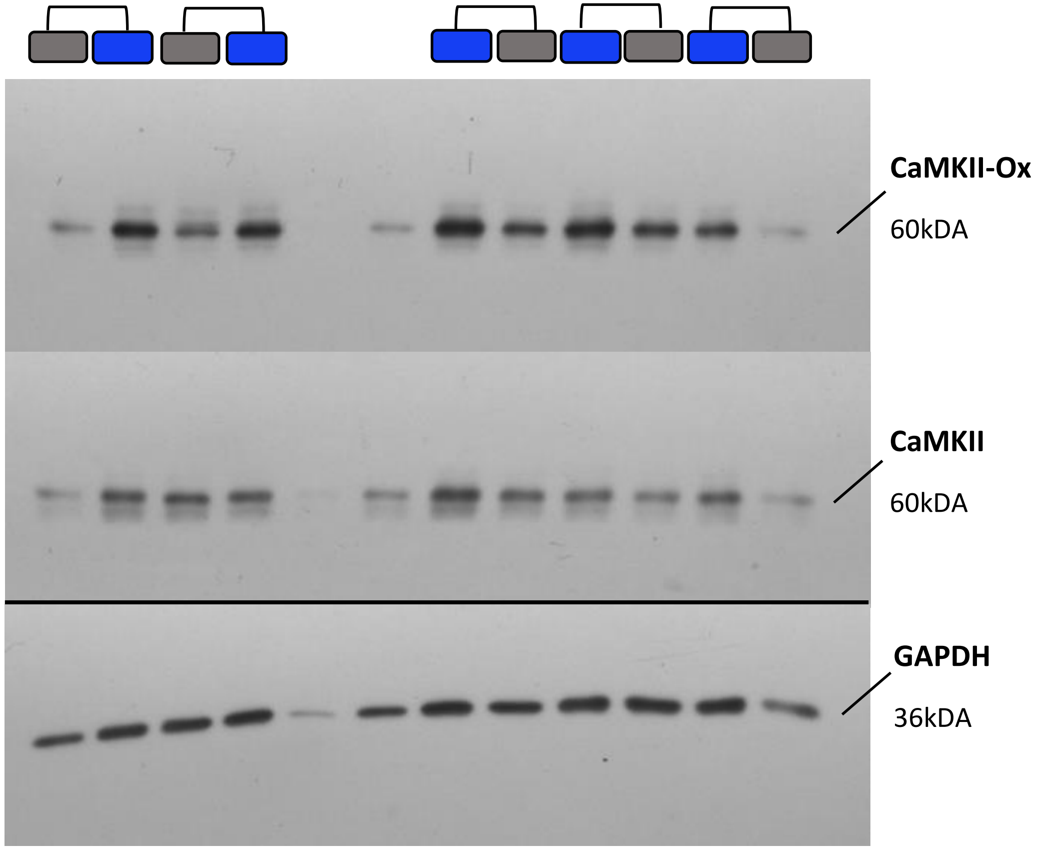

Control   AF simulation

Gel 2

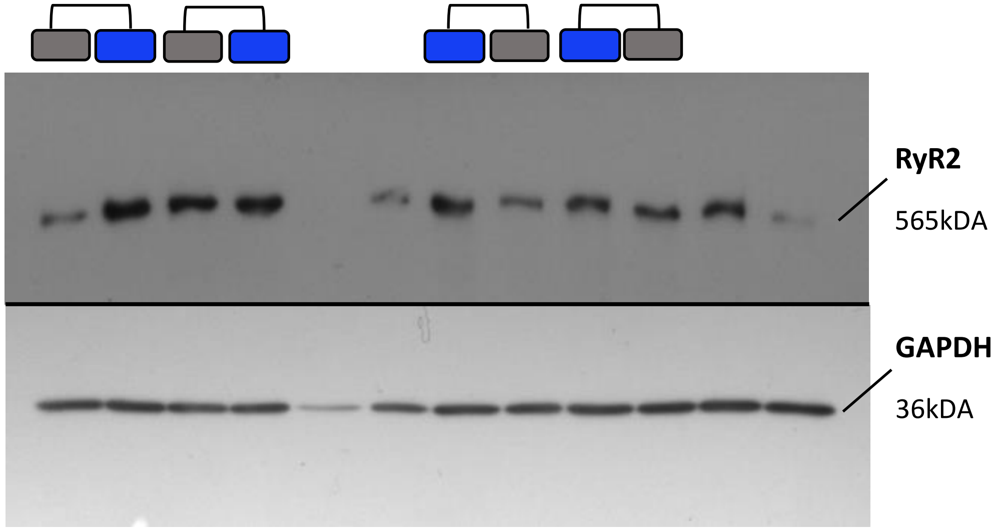

Gel 3

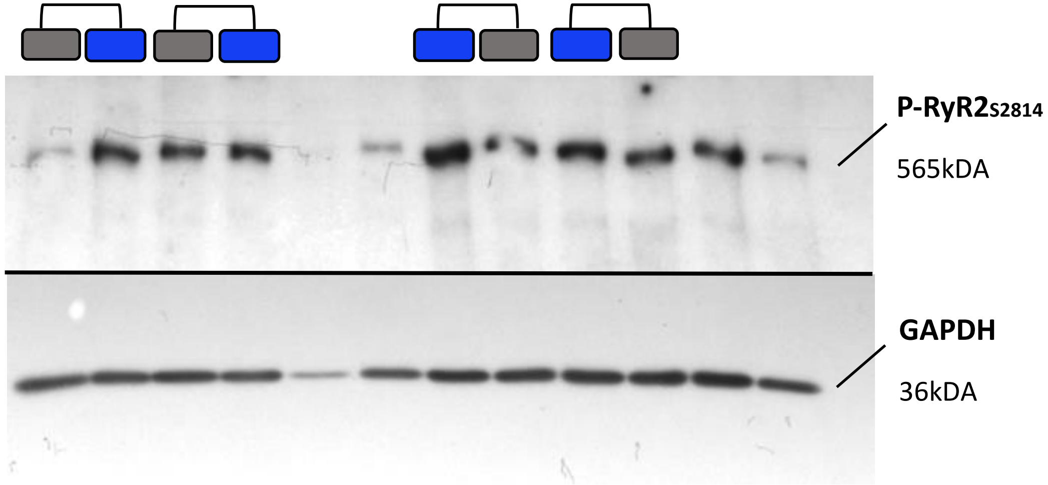

Blot 6

Gel 1

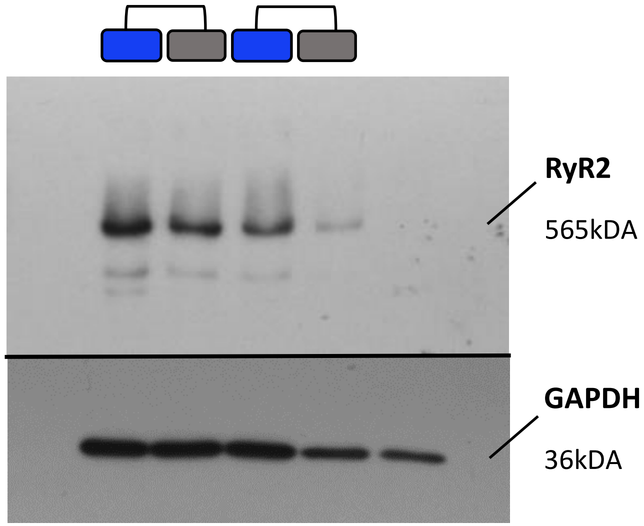

Gel 2

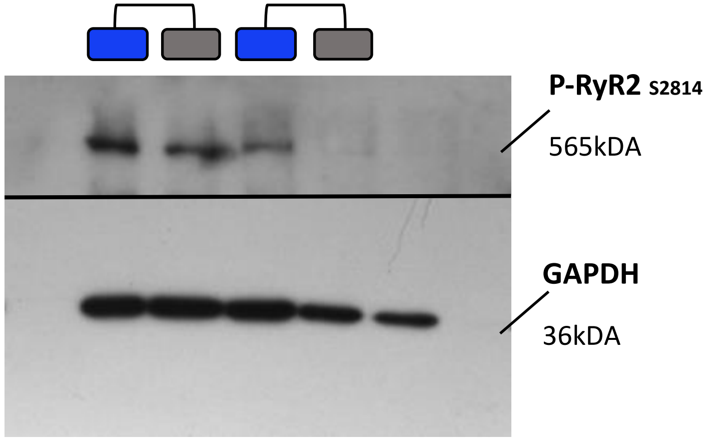

Blot 7

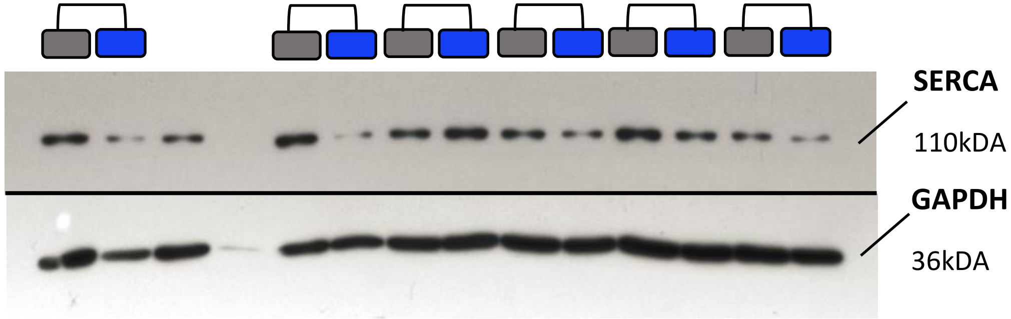

Control   AF simulation
